# A Comparison of Cranial Cavity Extraction Tools for Non-contrast Enhanced CT Scans in Acute Stroke Patients

**DOI:** 10.1007/s12021-021-09534-7

**Published:** 2021-09-06

**Authors:** L. Vass, M. J. Moore, T. Hanayik, G. Mair, S. T. Pendlebury, N. Demeyere, M. Jenkinson

**Affiliations:** 1 Wellcome Centre for Integrative Neuroimaging, Nuffield Department of Clinical Neurosciences, University of Oxford, Oxford, UK; 2 Department of Experimental Psychology, Radcliffe Observatory Quarter, Oxford, UK; 3 Centre for Clinical Brain Sciences, University of Edinburgh, Edinburgh, UK; 4 Neuroradiology, Department of Clinical Neurosciences, NHS Lothian, Edinburgh, UK; 5 Wolfson Centre for Prevention of Stroke and Dementia, Nuffield Department of Clinical Neurosciences, University of Oxford, Oxford, UK; 6 Departments of Medicine and Geratology and the NIHR Oxford Biomedical Research Centre, Oxford University Hospitals NHS Foundation Trust, Oxford, UK

**Keywords:** Computed tomography, Image segmentation, Brain extraction, Intracranial volume, Validation

## Abstract

Cranial cavity extraction is often the first step in quantitative neuroimaging analyses. However, few automated, validated extraction tools have been developed for non-contrast enhanced CT scans (NECT). The purpose of this study was to compare and contrast freely available tools in an unseen dataset of real-world clinical NECT head scans in order to assess the performance and generalisability of these tools. This study included data from a demographically representative sample of 428 patients who had completed NECT scans following hospitalisation for stroke. In a subset of the scans (*n* = 20), the intracranial spaces were segmented using automated tools and compared to the gold standard of manual delineation to calculate accuracy, precision, recall, and dice similarity coefficient (DSC) values. Further, three readers independently performed regional visual comparisons of the quality of the results in a larger dataset (*n* = 428). Three tools were found; one of these had unreliable performance so subsequent evaluation was discontinued. The remaining tools included one that was adapted from the FMRIB software library (fBET) and a convolutional neural network- based tool (rBET). Quantitative comparison showed comparable accuracy, precision, recall and DSC values (fBET: 0.984 ± 0.002; rBET: 0.984 ± 0.003; *p* = 0.99) between the tools; however, intracranial volume was overestimated. Visual comparisons identified characteristic regional differences in the resulting cranial cavity segmentations. Overall fBET had highest visual quality ratings and was preferred by the readers in the majority of subject results (84%). However, both tools produced high quality extractions of the intracranial space and our findings should improve confidence in these automated CT tools. Pre- and post-processing techniques may further improve these results.

**Supplementary Information:**

The online version contains supplementary material available at 10.1007/s12021-021-09534-7.

## Introduction

In research, extraction of the cranial cavity from neuroimaging scans is an important pre-processing step which is usually completed prior to further quantitative analyses. In many applications, additional tissue segmentation is applied to extract the brain tissue from other intracranial tissue such as cerebral spinal fluid (CSF), and measurements of intra-cranial volume are also useful for normalisation purposes. In MR analysis, tissue segmentation, image registration, and diagnosis often rely on brain extraction techniques (de Boer et al., 2010; Fennema-Notestine et al., 2006; Klein et al., 2010). It is therefore critically important to develop robust, effective, and externally validated techniques for performing brain extraction. Myriad brain extraction tools (BETs) have been developed and rigorously validated for MRI brain scans (Wang et al., 2014). In comparison, there is a paucity of equivalently well validated tools that extract the intracranial space for CT head scans. The wide availability, tolerability and low cost of CT has led it to becoming established as a mainstay of neuroimaging, particularly within acute clinical settings. Ideally, these tools would be fully automated, accurate and generalisable across the range of CT scanners while being able to cope with pathologies and image artefacts that are common amongst clinical and research CT scans.

A handful of automated tools have been developed to segment the intracranial space from non-contrast enhanced CT scans. Often the segmentation is colloquially referred to as brain extraction; however, the segmentations will include ventricular and subarachnoid CSF, meninges and other non-brain tissue. Therefore, it is more correct to say that these tools extract the cranial cavity (or intracranial space). Nevertheless, to maintain consistency with the previous literature, the term brain extraction tools (BETs) will subsequently be used throughout this paper. In two previous studies the MRI-based BET from the FMRIB software library (FSL; (Smith, 2002)) was independently adapted for CT scans. However, neither of these studies provided validation against a ground truth (Rorden et al., 2012; Solomon et al., 2007). Building on this, a subsequent study investigated the optimal settings for an FSL adapted tool and provided a rigorous validation (Muschelli et al., 2015), the authors implemented the tool using the publicly available R open-source program. A high accuracy of the resulting extractions was found (>99%) when compared to ground truth with dice similarity coefficient values exceeding 0.98. Another tool that has been validated was based on a particulate filter and was compared to manual delineations, but this tool was not fully automated (Jason G. Mandell et al., 2015). Besides the adapted FSL BET, two other automated tools have been recently developed for use in NECT. A deep learning model, based on a convolutional neural network (CNN), was trained and evaluated in stroke patients. Comparison with manual delineations revealed the tool produced brain extractions with high accuracy and precision. Further, dice similarity coefficient values were improved compared to the adapted FSL BET (0.998 vs 0.995) and the CNN-based tool had a substantially reduced execution time (Akkus et al., 2020). However, data were collected from a single centre and CT scanner, so it is unclear whether the tool will generalise to CT scans acquired under different conditions. The CNN-based tool is implemented in the freely available Python programming language and Keras deep learning framework. An additional tool, based on contour evolution with a novel propagation framework, has also shown promising results in non-contrast enhanced CT and CT angiography scans from stroke patients, again outperforming the adapted FSL BET approach based on dice similarity coefficient values (0.965 vs 0.877)(Najm et al., 2019). To further evaluate the robustness of the contour evolution tool, the investigators visually evaluated the results of their own tool in a large cohort of 1331 scans drawn from multiple-centres using CT scanners from several vendors. The investigators found a high degree of accuracy and a low failure rate (0.5%) even in the presence of image artefacts. Yet, they did not directly compare these against the quality of the brain extractions from the adapted FSL BET. The contour evolution tool is implemented in Matlab (MATLAB, 2010) and is also publicly available to researchers. Therefore, despite the recent progress in CT brain extraction, it is still challenging for a prospective researcher to distinguish the differences in performance of these tools and gauge the generalisability of these existing methods.

In this study, we sought to compare all freely available, automated BETs for use in non-contrast enhanced scans. We independently assessed these tools in an unseen cohort of real-world clinical CT head scans with varying image quality acquired from multiple scanners and evaluated their performance, ease of use and failure rate. In addition, we visually evaluated the performance of all the brain extraction tools on a regional basis, to identify the location of common errors in the brain extractions. Further, we aimed to identify post-processing techniques which may help to improve the results.

## Materials and Methods

### Participants

This investigation presents a retrospective analysis of a sub-set of routinely collected clinical CT scans from patients as part of cognitive screening studies led by ND (OCS-Tablet, OCS-Recovery, and OCS-CARE studies - NHS REC references 14/LO/0648, 18/SC/0550, and 12/WM/00335) between 2012 and 2020. These studies recruited consecutive stroke survivors, regardless of lesion location or behavioral pathology, during acute hospitalization. OCS-Tablet and OCS-Recovery recruited only within Oxford (e.g. (Demeyere et al., 2015, 2016)), though OCS-CARE recruited from 37 different stroke hospitals throughout the UK (Demeyere et al., 2019). No scans were excluded due to motion or beam hardening artefacts.

### CT Tools and Image Analyses

The literature was searched for BETs that had been developed or adapted for non-contrast enhanced CT scans. Existing tools were included for evaluation provided they were fully automated, were freely available at the time and had been used in at least one study that was published within the last 10 years. We searched the following databases: Embase, Medline, PubMed, SCOPUS and Web of Science for terms including CT or NECT, automated, brain, extraction, BET, segmentation, and delineation. Github was also searched using the same terms to identify available software. Besides conversion to the NifTI-1 format, no pre-processing of the CT scans was performed prior to their use in the tools.

Three brain extraction tools met our criteria for evaluation, the tools are summarised in Table 1 along with information on previous validations. Herein, we refer to the convolution neural network (CNN)-based tool as rBET (robust-BET), the contour evolution-based tool as cBET (as in contour-BET) and the FSL adapted tool as fBET. In brief, rBET is based on a 2D U-Net CNN with five encoder-decoder architecture, trained on 122 CT head scans of stroke patients from a single centre. cBET identifies the initial axial slice with the largest estimated brain cross section, then uses a convex optimisation algorithm based on a fully time-implicit level set scheme with global optimisation to propagate the brain outline. fBET models the brain surface by triangular tessellation and through an iterative process allows the surface to grow to find the optimal brain outline. More details on these algorithms are provided in the supplemental information. The tools were used as specified in the available documentation or appropriate publications. Since the fractional intensity (FI) parameter in fBET was evaluated at different values previously, we chose 0.01 based on experience and previous work (Muschelli et al., 2015; Najm et al., 2019). The average run time was calculated from 10 scans with different slice thicknesses, using an i7 core 2.5GHz CPU.

**Table 1:** Summary of the CT brain extraction tools included in this study

Tool name	fBET	rBET	cBET
Reference	Muschelli et al., 2015 [10]	Akkus et al., 2020	Najm et al., 2019 [15]
Base Method	FSL BET	CNN	Level-set evolving contour
Implementation and availability	Requires R and FSL - free	Requires Python and Keras - free	Requires Matlab subscription
Validation subjects			
*Quantitative (n)*	36	22	20
*Visual (n)*	129^*^	–	1337
Demographics of validation subjects^+^	Age**: 60.6 (11.6) Sex: 67% male	None provided	Age^***^: 73 (67–81) Sex: 60% NIHSS score***: 17 (12–19)
Inclusion criteria for trials in visual assessment^++^	Age: 18–80 Supratentorial ICH (>20 mL)	N/A	age >18^****^ acute ischemic stroke less than 12 h from symptom onset baseline NIHSS >5 ASPECTS >5
Comparisons with other tools	None	fBET, ITK skull stripping (Bauer et al., 2013)	fBET, ITK skull stripping (Bauer et al., 2013)

^*^Longitudinal data, the number of scans analysed was 1095

^**^Mean (standard deviation)

^***^Median (interquartile range)

^****^Summarises 3 trials, age >40 applied INTERRSECT trial only (Menon et al. 2018) baseline NIHSS and ASPECTS applied to ESCAPE trial only (Demchuk et al., 2015)

^+^These only apply to the subjects with quantitative results

++ Reduced version of inclusion criteria

*ASPECTS* Alberta stroke programme early CT score, *BET* brain extraction tool, *CNN* convolution neural network, *FSL* FMRIB software library, *n* number of subjects, *ICH* intracerebral haemorrhage, *NIHSS* National Institutes of Health Stroke Scale

### Quantitative Comparison to Ground Truth

Manual cranial cavity delineation was performed on 20 randomly selected CT head scans from the full dataset of 428 scans. An experienced neuroimaging researcher (MJM) employed MRIcron to outline the intracranial space; voxels within the intracranial space were assigned a value of 1, all other voxels were assigned a value of zero, thus a binary mask was produced for each of the 20 subjects. Although this binary mask does not represent brain tissue alone, we use the term binary mask and brain mask interchangeably, to maintain consistency with previous publications. The brain masks were approved by an experienced neuro-radiologist (GM). The binary masks produced by the BETs were used in all subsequent analyses. To assess performance of the tools we calculated the dice similarity coefficient (DSC), accuracy, precision and recall. The DSC measures similarity between two sets of data at a voxel level; it can be defined as $DSC=\frac{2 TP}{2 TP+ FP+ FN}$ where TP, FP and FN represent true positives, false positives and false negatives, respectively.

As a complementary method to assess the regional differences between the two tools, average voxel-wise FP and FN maps were generated. Each subject’s BET masks were compared to the ground truth mask to produce a spatial map of FP voxels and a map of FN voxels. Next, each subject’s CT scan was registered to a common space using an affine registration to an age appropriate CT head template (i.e. mean age 65 years old) (Jenkinson et al., 2002; Jenkinson & Smith, 2001). For each of the 20 subjects, the FN and FP maps were subsequently registered to this common space using the calculated affine transformation matrix. Since all the FP and FN maps were now aligned to a common space, we could calculate the mean number of FN and FP voxels.

### Qualitative Assessment

Qualitative, visual assessment of the brain masks was performed for all 428 CT brain scans by three experienced neuroimaging researchers (LV, MJ, MJM). To provide information on both the global and regional BET performance, each brain mask was assessed in 11 different regions (see Table 2). These regions were chosen either due to the expectation for the region to be a common failure point (e.g. foramina) or because the region was considered an area where accuracy may be important for research outcomes (e.g. temporal lobes, cerebellum). Each BET’s performance was rated on a scale of 1–4: 1 = excellent, 2 = good, 3 = intermediate and 4 = failure. To minimise inter-rater variability, all assessors rated a small test subset of brain masks to harmonise ratings before proceeding to the full scan set. These test ratings were not included in the results.

**Table 2:** Description of regions for visual assessment

Reference region	Abbreviation	Description
Overall		Overall quality of the extraction
Pituitary fossa	PF	Errors in mask within the PF
Petrous temporal bone	PTB	Mask within the inner skull table
Internal auditory meatus	IAM	Errors in mask within the internal auditory canal
Olfactory bulb	OB	Mask within the inner skull table
Cerebellum	CB	Included, including tonsillar descent
Temporal lobe	TL	Both lobes included in entirety of mask
Frontal lobe	FL	Included in entirety of mask
Extracranial	EC	Regions outside of skull excluded
Intracranial	IC	Errors in mask within skull (not covered by other categories)
Skull defect	SD	Mask continuous with inner table of skull in presence of defects

### Statistics

Bland-Altman (BA) plots were used to compare the automated brain extractions to the ground truth manual delineations. The mean difference in intracranial volume (ICV) between the BET masks and the corresponding ground truth masks is reported along with the limits of agreement (mean ± 1.96 x standard deviation of the differences). The assumption of normally distributed differences was tested using the Shapiro-Wilks test. Median, first and third interquartile values are shown in boxplots. Paired t-tests were used to assess significant differences in performance metrics. Unless otherwise stated, *p* < 0.05 was considered significant.

## Results

### Overall Comparison

The demographics and characteristics of the patients from whom CT head scans were obtained are summarised in Table 3. The scans included were from 28 different sites representing a diverse set of CT scanners and image characteristics. The three BETs were evaluated in 428 CT head scans: fBET produced an output mask for all patient scans, rBET did not produce an output mask in 4% of patient scans, cBET did not produce an output mask in 63% of the scans. The average run time per scan for fBET, rBET and cBET was 142 ± 35 s, 20.5 ± 3.8 s and 16.4 ± 4.5 s. Given the unexpectedly high failure rate of cBET, data from this tool was not included in subsequent analyses (more information regarding this decision is provided in the Discussion section).

**Table 3:** Demographics and characteristics of patients with CT head scans used as inputs to the brain extraction tools

Number of subjects		428
Age (mean)		71.3 ± 13.3
Sex (% Male)		57
Number of hospital sites		28
Axial slice thickness	<1 mm	2%
	3*mm* > *x* ≥ 1*mm*	24%
	5*mm* > *x* ≥ 3*mm*	8%
	≥5 mm	66%
	5.3 mm	maximum

### Comparison with Ground Truth

**Fig. 1:**
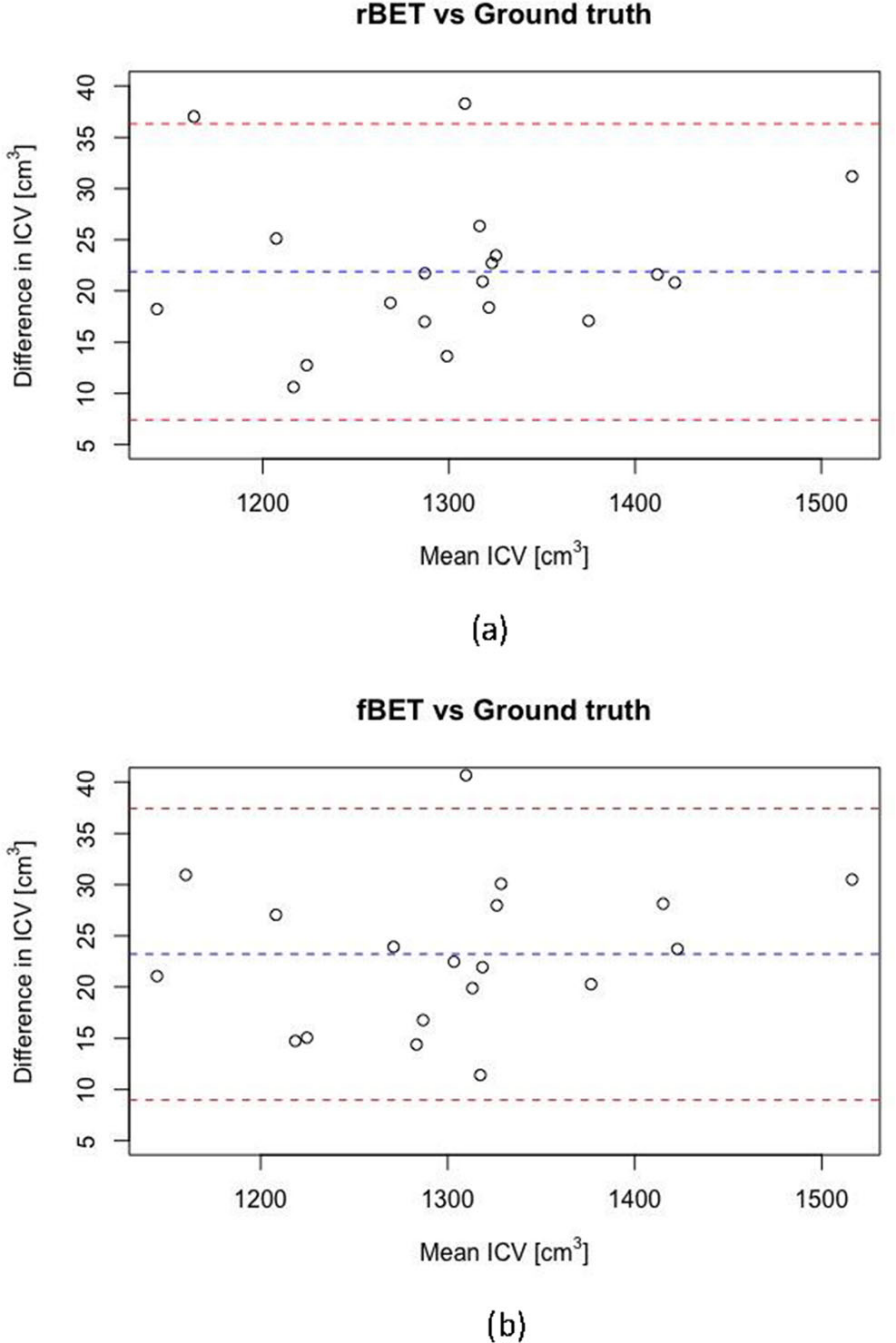
Bland Altman plots comparing intracranial volume (ICV) measured using automated CT tools to ICV derived from manual delineations (considered ground truth). In (**a**) ICV measured using rBET is compared to ground truth and (**b**) shows ICV measured using fBET compared to ground truth. *The blue dotted lines indicate the mean, red dotted lines indicate upper and lower limits of agreement. ICV = Intracranial volume, fBET = FSL adapted tool brain extraction tool, rBET = CNN-based brain extraction tool*

The Bland-Altman plots for fBET and rBET are shown in Fig. 1, data were normally distributed according to the Shapiro-Wilks test (*p* = 0.68 and 0.54, respectively) and there was no statistically significant linear association between the differences and the mean. The mean difference in ICV between fBET and the manual delineation was 23.2 ± 7.3 cm^3^; the limits of agreement were 9.0 and 37.5cm^3^. This mean difference is equivalent to 1.79% of the average ICV of 1291cm^3^. Similarly, the mean difference in ICV between rBET and ground truth was 21.9 ± 7.4 cm^3^ (or 1.69% of the average ICV); the limits of agreement were 7.41 and 36.3cm^3^ Both measurements show evidence of bias by over-estimating the volume compared to the manual delineations.

**Fig. 2:**
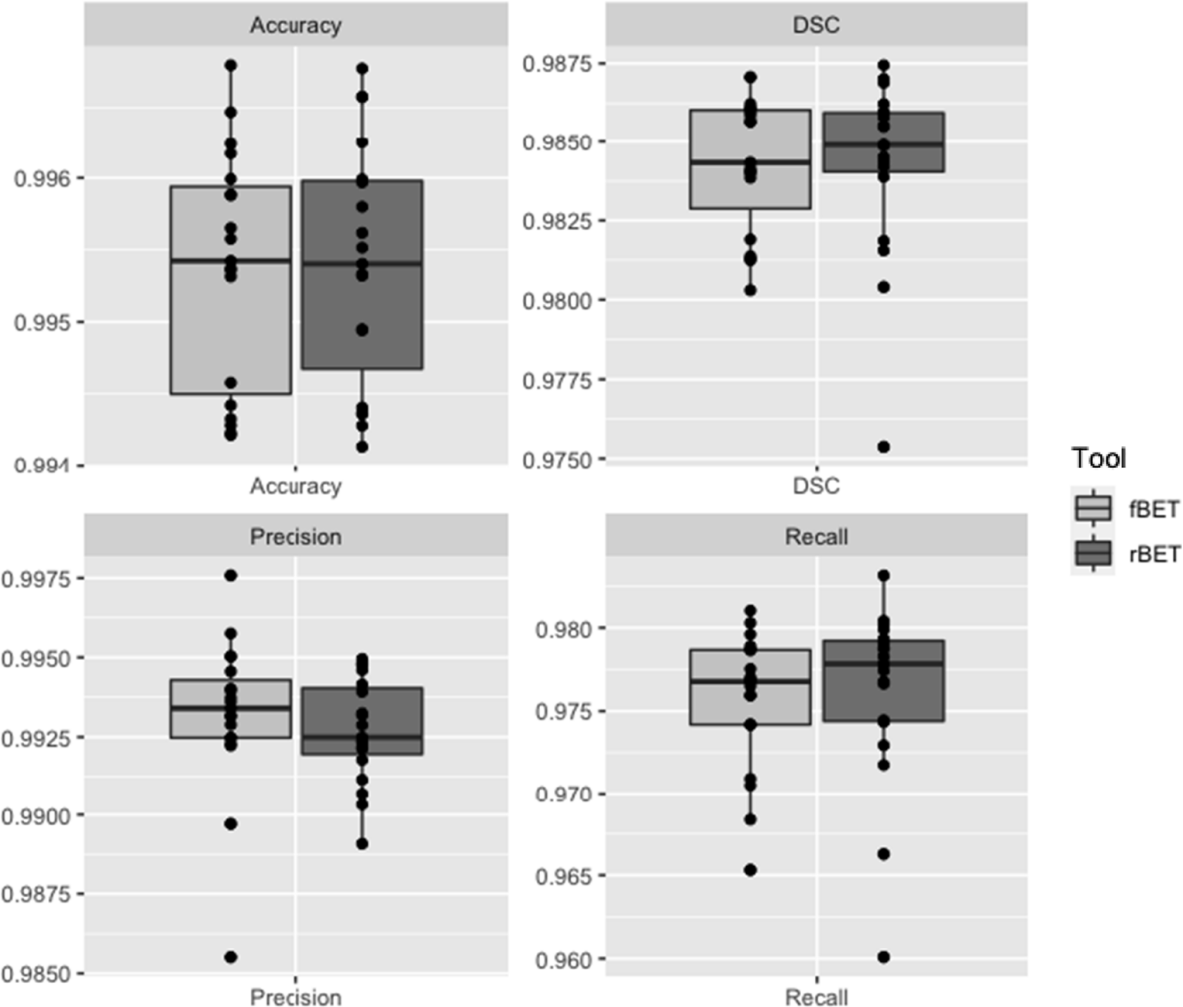
Comparison of performance metrics using CT brain extraction tools in 20 patients. *DSC = Dice similarity coefficient, fBET = FSL adapted tool, rBET = CNN-based tool*

The accuracy, precision, recall and DSC values are summarised in Fig. 2. The performance metrics were excellent and comparable between the two tools: the mean accuracy, precision and recall were indistinguishable (fBET: 0.995 ± 0.001, 0.993 ± 0.003, 0.976 ± 0.004; rBET: 0.995 ± 0.001, 0.993 ± 0.002, 0.976 ± 0.005; *p* = 0.86, *p* = 0.47, *p* = 0.46, respectively). Equally, the mean DSC values were equivalent between the two tools (fBET: 0.984 ± 0.002; rBET: 0.984 ± 0.003; *p* = 0.99).

**Fig. 3:**
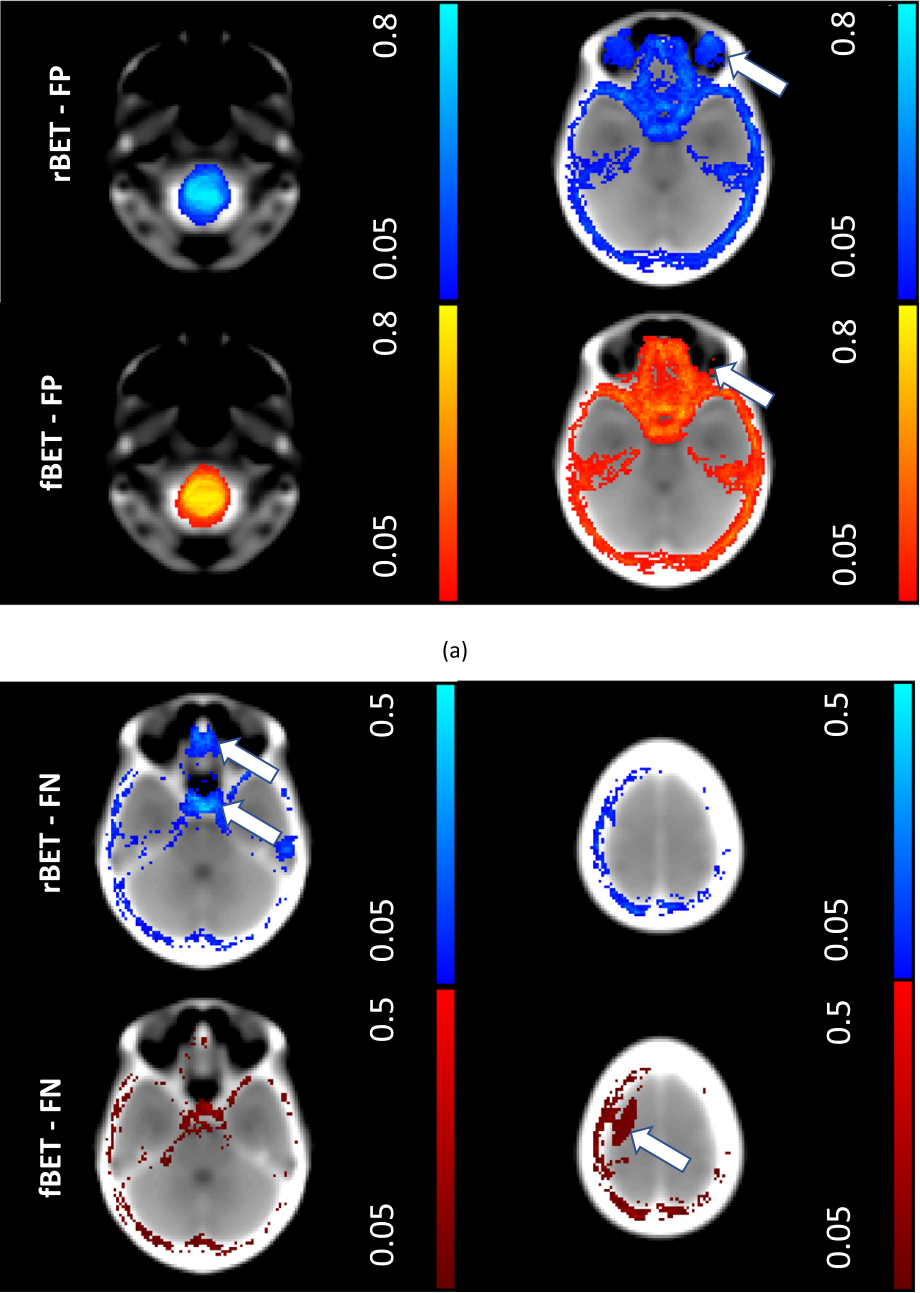
Average false positive and negative voxel wise maps for CT brain extraction tools in 20 subjects. Multiple axial slices are shown highlighting interesting features in the brain extractions. In (**a**) the top row is the average FP map using rBET, the bottom row shows the average FP map in the corresponding slice using fBET. Shown is the propensity for both rBET and fBET masks to include the brain stem (left hand side, *LHS*) and on the right (RHS) the eyes are included in rBET (see arrows). In (**b**) the top row is the average FN map using rBET, the bottom row shows average FN map in the corresponding slice using fBET. The LHS shows the tendency for rBET to exclude the pituitary gland and part of the frontal lobe, the RHS shows that fBET missed a portion of the parietal lobe. *Each individual subject map was registered to standard space using a CT template. The scale is indicated on the RHS: a value of 0.05 is equivalent to a FN or FP for one single patient at that voxel position. FN = false negative, FP = false positive, LHS = left hand side, RHS = right hand side*

Clear differences were noted between the tools from their respective FN and FP maps (see Fig. 3). rBET tended to exclude brain tissue in the pituitary fossa and inferior frontal lobe. In two patients, fBET excluded part of the parietal lobe. The FP maps reveal that rBET had a predilection to include tissues within the eye socket, fBET tended to include tissues in the olfactory bulb region in the mask. In common, both tools were more likely to produce FPs rather than FNs. Another common feature of both tools was the inclusion of tissue beyond the lower margin of our manual delineations; chosen as 5 mm inferior to the foramen magnum to allow for normal cerebellar tonsillar descent.

### Qualitative Comparison

**Fig. 4:**
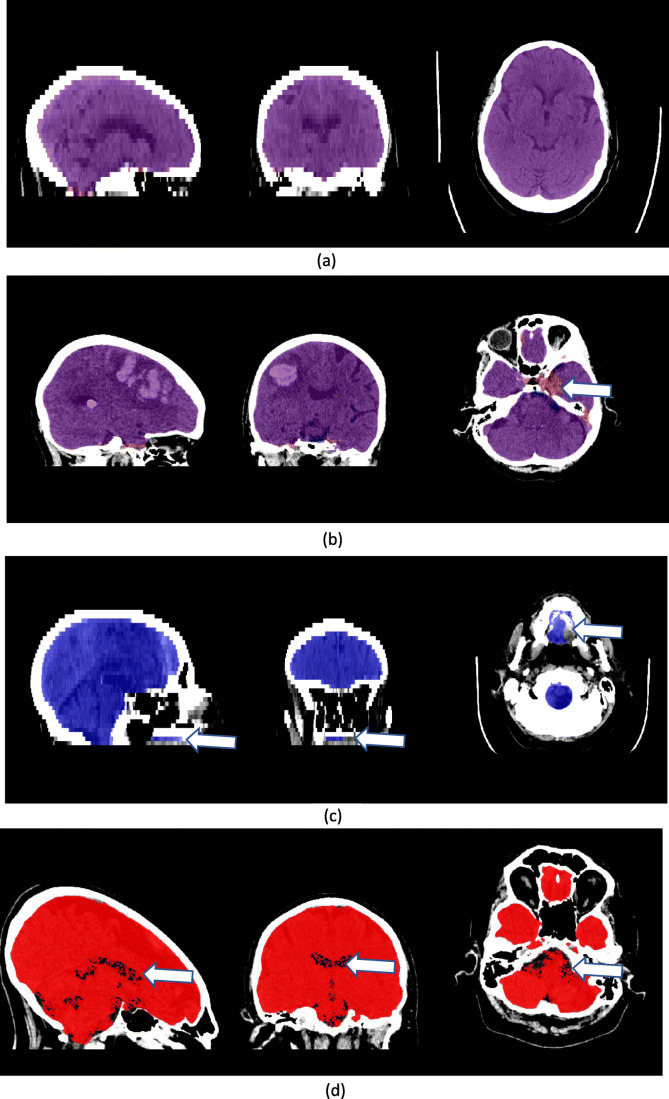
Examples of the brain masks from automated tools used in the visual assessment: (**a**) shows an example of excellent quality for the fBET and rBET masks. In (**b**) the inferior portion of the temporal lobes are partially missing in the brain mask using rBET (indicated by arrows), for comparison the fBET mask has performed better. In (**c**) the rBET mask includes tissue inferior to the hard palate, i.e. in the roof of mouth, otherwise the quality is excellent. (**d**) provides an example of the fBET mask containing a series of internal holes. *Red = brain mask using fBET, blue = brain mask using rBET, (a) and (b) have the rBET mask overlaid on the fBET mask, so it is purple where they agree*. *From left to right: sagittal, coronal and axial slices are shown*

Table 4 summarises the average visual ratings and the proportion of scans that received each rating for global and regional BET performance. In general, the majority of errors occurred in the base of the brain for both tools. The average rating for overall performance was considered ‘good’ for both fBET and rBET; however, fBET had fewer scans with a rating of intermediate (11% vs 34%) and more with an excellent rating (12% vs 2%; see Fig. 4a).

**Table 4:** Average visual quality ratings of brain extraction tool results. Overall, the brain extractions using fBET were preferred in 83% of the subjects

	Average rating		% with rating 1		% with rating 2		% with rating 3		% with rating 4	
	fBET	rBET	fBET (%)	rBET (%)	fBET (%)	rBET (%)	fBET (%)	rBET (%)	fBET (%)	rBET (%)
Overall	2	2.4	12	2	75	61	11	34	3	4
TL	1.4	2.2	59	13	40	58	0	25	0	4
FL	1.4	1.6	65	51	34	42	0	7	1	0
CB	1.7	1.8	32	39	66	46	2	11	0	4
PF	1.7	2.7	40	11	49	35	9	28	2	26
PTB	1.9	1.7	29	41	57	47	14	12	0	0
IAM	1.8	1.4	30	64	63	31	6	5	0	0
OB	1.7	1.9	44	33	46	47	9	15	1	5
SD^*^	3.0	2.0	0	0	0.14	0.5	0.35	0	0	0
EC	1.9	2.6	32	18	47	29	15	25	6	27
IC	1.6	1.5	45	55	49	40	3	5	4	1

Ratings: 1 = excellent, 2 = good, 3 = intermediate, 4 = failure

*fBET* = FSL adapted tools, *rBET =* CNN based tool

^*^Only 2 scans had skull defects so reported percentages are small

Regional comparison of the results in the petrous temporal bone, cerebellum and olfactory bulb found similar ratings for the tools, the majority were either excellent or good. However, there were characteristic features of both tools that resulted in different performances in certain regions.

Brain extraction using fBET had a higher quality rating in the frontal and temporal lobes compared to rBET. This was due to the tendency for rBET to underestimate the frontal lobes, typically missing the inferior axial slice(s). Equally, rBET underestimated the temporal lobes (see Fig. 4b), this resulted in 25% of scans receiving intermediate quality ratings in this region whereas using fBET only excellent or good ratings were given in this region. There was also a tendency for rBET to incorrectly include small regions outside the skull including the eyes, tongue and throat (see Fig. 4c); this resulted in poorer quality in the extracranial category and a large proportion of failures (27%). Notably, rBET failed in the pituitary fossa region in a much larger proportion of cases than fBET (26% vs 2%); in these cases, the tool failed to include the pituitary gland (see Fig. 4b).

Conversely, rBET performed on average better than fBET in the region of the internal auditory meati and skull defects. There was a tendency for the brain mask produced by fBET to spill into the ear canals; this resulted in an excellent rating only occurring in 35% of subjects compared to 65% when using rBET. Skull defects were rare in these data (only 2 subject scans) but fBET spilled out of any holes in the skull whilst brain masks using rBET remained within the expected inner skull line. In addition, the consistency of the brain was better using rBET (i.e. the internal category in Table 4), due to the tendency of fBET to contain multiple small holes in the brain masks. These holes appear in regions of negative CT numbers; typically, in cisterns and ventricles with values between −10 – 0 HU, this is likely due to drift in the CT scanners calibration (Fig. 4d shows one of the more pronounced examples). Importantly, despite similar overall ratings, the brain mask produced by fBET was preferred over the one produced by rBET in 83% of the scans.

## Discussion

In this study, we independently tested CT brain extraction tools on a large number of clinical quality non-contrast enhanced CT head scans; scans acquired from a large, diverse, and representative cohort of acute stroke patients. Overall performance in all but one of the tools was comparable with high levels of accuracy, precision and DSC values compared to the ground truth. However, intracranial volume was overestimated using the tools due to inclusion of tissue inferior to the foramen magnum. Although it was difficult to distinguish differences between the tools using quantitative metrics alone, we identified clear regional differences in the resulting brain masks which may be prove helpful to guide future investigators requiring brain extraction in CT scans.

We identified three BETs that were designed specifically for non-contrast enhanced CT: rBET, fBET and cBET. However, we decided not to include cBET in the final analysis due to the high number of extraction failures. This high failure rate was unexpected given the extensive validation of this tool in 1331 non-contrast enhanced CT scans gathered from multiple centres (Najm et al., 2019). In the present study, cBET extraction failures were not associated with obvious potential scan variations such as image quality or gantry tilt. The source code is not made available to users, and so this obfuscation limited our ability to further investigate the failures. We contacted the authors but were unable to perform further investigations with their help due to a combination of restrictions around intellectual property and ethics requirements. Whilst it is not possible to make firm conclusions about cBET, excellent quality was generally observed in those subjects with completed brain extractions. Additional research is needed to further understand the high failure rate found in our data.

We utilised the tools as specified, with no additional pre-processing or manipulation of the input CT images. This lack of pre-processing is an advantage as it makes the extraction tools more straightforward to implement on a diverse range of datasets. As research tools, both rBET and fBET were well packaged and easy to install and run, although both require a basic familiarity with executing code using a command line interface. If brain extraction is required on a large numbers of scans, rBET was found to be favourable due to the reduced execution time.

Comparison of the resulting BET masks with manual delineation revealed equivalent results for rBET and fBET across performance metrics. This demonstrates that both tools appear to generalise well. This is reassuring for deployment of rBET, given that the training data used to develop rBET was obtained solely from one site and the lack of information surrounding the diversity of their clinical characteristics. Other investigators observed lower DSC values (= 0.877 ± 0.038) when using fBET (Najm et al., 2019); however our results show higher values that are consistent with other reported DSC values for fBET (i.e. DSC = 0.9895 ± 0.002 (Muschelli et al., 2015) and 0.995 ± 0.002 (Akkus et al., 2020)).

Bland-Altman analysis demonstrates that the tools gave comparable agreement with the ground truth when estimating intracranial volume. Yet, the agreement is notably poorer than the previously reported values (Akkus et al., 2020), exhibiting an overestimation bias. This bias is likely due to the difference in inferior margin of the mask: our manual delineations terminated 5 mm inferior to the foramen magnum whereas the automated tools overestimated intracranial volume by extending the brain mask several centimetres further. In future studies, end-users should be careful near the base of the brain as we observed inconsistent stopping points among our data.

Despite the good quantitative agreement between fBET and rBET masks, the false negative and false positive maps and visual comparisons identified consistent regional differences in tool performance. Analysis of false positive maps revealed that rBET was more likely to incorrectly include tissues in the eye sockets and tissues of the throat than fBET. This was consistent with the results of the visual evaluation as rBET had a larger proportion of failures and a lower rating of intermediate quality in this category. Nevertheless, these false positive regions could be readily excluded using a post-processing procedure such as a connected components algorithm (Preim & Botha, 2014). In addition, rBET was more likely to exclude the pituitary gland and parts of the inferior temporal lobes than fBET. If consistent extraction of the pituitary gland is required, then this tool may be unsuitable. In comparison, fBET tended to erroneously include tissues near the olfactory bulb, the ear canals and extended outside the skull when defects were present; rBET performed better in these regions. On rare occasions, fBET missed substantial proportions of the brain due to self-intersection, resulting in failure. More generally, it was noted from the visual assessments that there were small holes in fBET masks, typically only a few voxels and these were coincident with voxels that had negative CT intensity values. fBET was adapted from its original purpose to extract MRI brain scans, since MRI scans do not have negative values, it is likely the program had not encountered the negative values seen in CT scans. However, this was a very minor issue and could be easily resolved using pre-processing. Overall, the visual comparison demonstrated that the brain extractions using fBET were decidedly preferred by the readers (i.e. in 83% of the scans). Interestingly, in the 27% of cases where the rBET brain extractions were preferred, ratings in the temporal lobes and extracranial tissues were more comparable to the fBET tool, indicating that if the performance of rBET could be consistently improved in these regions then future investigators may find that overall preference is more equivalent than reported here.

The observed performance differences between the tools can be accounted for by differences in the underlying methods. Given the black-box nature of the deep-learning rBET algorithm, only limited understanding of this approach’s exact underlying method is possible. However, the observed results of rBET may be partly driven by the training data. For example, rBET often failed to completely capture the pituitary gland and the inferior portion of the temporal lobes. The appearance of these regions are more heterogenous between patients’ scans than, for example, the parietal lobe due to prominent differences in skull shape and head angles. Given that a comparatively small training set from a single institution was used to develop rBET, it is possible that the training data were insufficiently diverse to provide accurate predictions in these regions. In contrast, fBET relies on constructing an optimal brain surface through an iterative process, and therefore demonstrated a tendency to overestimate the intracranial space caused by surface spilling over near the olfactory bulb and ear canals. With fBET it is possible to control the size of the final mask by manual optimisation of parameters e.g. increasing the fractional intensity (option ‘-f’) will reduce the overall outline size, but the value of 0.01 produced the best quality of brain extractions in our experience and in previous studies (Muschelli et al., 2015; Najm et al., 2019).

In this study, we were able to assess CT brain extraction tools in real-world clinical data using different scanners with varying image quality. These scans are representative of the older patients in whom CT is a front-line imaging modality. We compared the results of each tool both against the gold standard of manual delineations and in detailed regional visual assessments. However, there were limitations in this study. We did not investigate the reproducibility of manual delineations on our data, however previous results indicate that 95% of the differences in intracranial volume between two experts lie within approximately −7 and 8 cm^3^ (Akkus et al., 2020) and with a mean difference in intracranial volume of 1.2% [8]. Accounting for the previously mentioned discrepancy in brain stem inclusion, these appear comparable to automated methods reported here. In addition, we applied these tools in stroke patient scans only - similar to previous studies, other patient cohorts may result in more pronounced differences in performance (e.g. neonates, craniotomy). Further, we did not investigate the difference in performance due to changes in the CT reconstruction kernel, which was found to be a factor in the performance of fBET (Akkus et al., 2020). However, our data was reconstructed using soft tissue reconstructions, which is advisable for CT brain studies. We could not assess the repeatability of the tools, it is hoped that future longitudinal studies will be able to assess repeatability. To produce the FN and FP maps we relied on registration to an age appropriate CT template which may introduce errors and hence there may be some mis-alignment in the position of the voxels in template space, although this would only mis-locate the errors and neither create nor suppress FPs or FNs. In addition, we performed quality control and visually compared original positions of the FN/FP voxels to those registered in template space; we noted no substantial difference in native and template space.

For clarity, it is worth reiterating that the tools reviewed in this study extract the intracranial space rather than purely the brain tissue, so are not truly brain extraction tools. This is useful for intracranial volume measurements, but subsequent processing of the intracranial masks is required to extract the brain tissue. To facilitate translation of such tools into clinical practice and further research endpoints, CT tools need to be robust, accurate and reliable. The relative importance of these will depend to a varying extent on the endpoint of the analysis. For example, in a longitudinal analysis of intracranial volume, repeatability will be of paramount importance, while as a pre-processing step prior to deep learning methods it may be less important. In our data, we found quantitative metrics alone led to difficulty in distinguishing the tools. Overall rBET and fBET exhibited high accuracy but our findings indicate that robust reporting of different features from visual assessment is also required to evaluate overall tool performance. Overall the masks produced by fBET were preferred but both tools seemed robust when generalised to unseen clinical quality scans of unselected stroke patients. Without large publicly available datasets of scans – as are readily available for MRI – validation of CT-based tools is limited by those datasets available at individual research and healthcare institutions. It is hoped that the results presented here will help to inform choices and improve confidence in the tools used in non-contrast enhanced CT head scans and aid future investigators in understanding the limitations and strengths of existing tools.

## Conclusion

We independently tested freely available, fully automated CT tools that extract intracranial space in an unseen dataset of 428 CT head scans from stroke patients. Although one CT extraction tool did not perform well on our data, we found high accuracy for the two remaining available tools, suggesting they are robust and generalisable when applied to CT head scans of stroke patients. Subsequent global and regional visual evaluation revealed that the independently adapted version of the FSL brain extraction tool was preferred in our dataset. Straightforward pre-processing and post-processing techniques may improve performance further.

## Information Sharing Statement

The datasets used for and generated during the analysis are available on reasonable request from the corresponding author upon completion of a material transfer agreement. The code is freely available (see Code Availability).

## Supplementary Information

ESM 1 (DOCX 22 kb)

## Data Availability

The datasets used for and generated during the analysis are available on reasonable request from the corresponding author upon completion of a material transfer agreement.

## Code Availability

All code used in this analysis is freely available, see references for code repositories.

## Declarations

The views expressed are those of the authors and not necessarily those of the NHS, the NIHR or the Department of Health and Social Care.
