# Supplementary material for: A Comparison of Cranial Cavity Extraction Tools for Non-contrast Enhanced CT Scans in Acute Stroke Patients
Source: Neuroinformatics. 2021 Sep 6;20(3):587–98. doi: 10.1007/s12021-021-09534-7 (PMC9547790; doi:10.1007/s12021-021-09534-7)
Supplement: Supplementary file 1 — (DOCX 22 kb) [file 12021_2021_9534_MOESM1_ESM.docx]

**Supplemental information**

The following supplementary material provides more details of the algorithms behind the CT brain extraction tools. The descriptions below provide an overview of the tools used in this study, further details including mathematical formulations can be found in the respective references. It is important to note that the tools were used as specified in the references and all default settings were not altered in our analyses.

**fBET (Muschelli *et al.*, 2015) - adapted brain extraction tool from the FMRIB software library (FSL)**

1. A threshold of 0-100HU (i.e. voxels outside this range are set to 0 HU) is applied to the input 3D head CT images.
2. Images are smoothed with a Gaussian filter using kernel size of σ = 1mm^3^ and then a threshold between 0-100HU is reapplied.
3. FSL BET is then applied with fraction intensity (option ‘-f’) set to 0.01. FSL BET performs the following steps (Smith, 2002):
   1. A histogram-based threshold estimation of ‘robust’ lower and upper intensity values yielding a rough brain/non-brain threshold.
   2. The centre-of-gravity (COG) and rough head size is estimated from the image
   3. The brain surface is modelled by triangular tessellation, initially generated as a tessellated sphere centered on the COG with a radius at half the estimated head radius.
   4. Through an iterative process the surface is allowed to grow to find the optimal estimate of brain outline, using forces based on intensity gradients, inflationary force along the normal, and within-surface forces to encourage even spacing of vertices.
4. Post-processing performed by hole filling (> 0 HU)

**rBET (Akkus *et al.*, 2020) - Convolution neural network (CNN)-based brain extraction tool**

1. No pre-processing of the input 3D head CT images is performed
2. A 2D U-Net convolutional neural network architecture is used which is equivalent to the best performing model in the original proposal (Akkus *et al.*, 2020).
3. The network consists of a five encoder-decoder CNN. The encoder consists of 10 convolution layers with 3 x 3 kernels. Followed by Maxpooling to downsample the output of the convolutional layers and the Rectified Linear Unit (ReLU) activation function. The decoding part consists of 9 convolution layers with 3 x 3 kernels and 4 deconvolutional upsampling layers to ensure the output mask maintains the same dimensions as the input CT image.
4. Network weights are initialised using the Glorot uniform distribution, the weights are updated using the Adam optimiser with an initial learning step of 10^-5^. The loss function is weighted categorical cross entropy with drop-out (probability of 0.5) applied twice in the encoding part of the network (following convolutional layers 8 and 10).
5. The final CNN layer is connected to a SoftMax output layer in order to make a prediction as to whether a pixel is classified as brain or non-brain.

**cBET (Najm *et al.*, 2019) - Contour evolution-based tool**

1. A threshold of 100HU is applied to the input 3D head CT images followed by smoothing with a Gaussian filter with a kernel size of σ = 1mm^3^. Connected component analysis is performed in each slice along the z-axis. The axial slice with the largest estimated brain cross section is identified; this is used as the initial slice.
2. 2D morphological operations and connected component analysis are used to segment the brain from the localised initial slice.
3. Segmentation in the initial slice is propagated to the two neighbouring slices (i.e. inferior and superior). The segmentation is reused in these adjacent slices both as an initial contour and a propagation shape constraint in the convex optimization segmentation algorithm.
4. The convex optimisation algorithm is based upon a fully time-implicit level set scheme with global optimisation. An additional term was proposed that penalises the difference between the evolved contour and the propagated contour (from previous slice) to ensure consistency of brain contours along the z-axis (superior-inferior direction).
5. Post-processing performed including hole filling and small island removal.
